# Environmental Tobacco Smoke Exposure Estimated Using the SHSES Scale and Epicardial Adipose Tissue Thickness in Hypertensive Patients

**DOI:** 10.1007/s12012-020-09598-y

**Published:** 2020-08-18

**Authors:** Paweł Gać, Karolina Czerwińska, Małgorzata Poręba, Piotr Macek, Grzegorz Mazur, Rafał Poręba

**Affiliations:** 1 Department of Hygiene, Wroclaw Medical University, Mikulicza-Radeckiego 7, 50-368 Wrocław, Poland; 2 Centre of Diagnostic Imaging, 4th Military Hospital, Weigla 5, 50-981 Wrocław, Poland; 3 Department of Pathophysiology, Wroclaw Medical University, Marcinkowskiego 1, 50-368 Wrocław, Poland; 4 Department of Internal Medicine, Occupational Diseases and Hypertension, Wroclaw Medical University, Borowska 213, 50-556 Wrocław, Poland

**Keywords:** Environmental tobacco smoke, SHSES scale, Hypertension, Epicardial adipose tissue, Computed tomography

## Abstract

The aim of the study was to assess the relationship between environmental tobacco smoke exposure (ETS) and epicardial adipose tissue thickness (EATT) in hypertensive patients. A total of 96 patients with essential hypertension were recruited for this study. The group consisted of 48 females and 48 males with the mean age of 69.32 ± 9.54 years. ETS was assessed with The Secondhand Smoke Exposure Scale (SHSES). EATT was assessed in 128-slice dual source coronary computed tomography angiography. In accordance to SHSES scale patients were divided into subgroups: subgroup A—no ETS exposure (SHSES = 0 points, *n* = 48), subgroup B—low ETS exposure (SHSES = 1–3 points, *n* = 11), subgroup C—medium ETS exposure (SHSES = 4–7 points, *n* = 20) and subgroup D—high ETS exposure (SHSES = 8–11 points, *n* = 17). Within the study group the mean EATT was 5.75 ± 1.85 mm and the mean SHSES score was 3.05 ± 3.74. EATT was statistically significantly higher in subgroup D than in subgroups A and B (A: 5.28 ± 1.64 mm, B: 5.04 ± 2.64 mm, D: 7.04 ± 2.64 mm, *p*_A–D_ and *p*_B–D_ < 0.05). There was a positive linear correlation between the exposure to ETS expressed by the SHSES scale and EATT (*r* = 0.44, *p* < 0.05). Regression analysis showed that higher SHSES score, higher BMI, and higher systolic and diastolic blood pressure are independent risk factors for higher EATT values. Contrary, the use of ACE inhibitors and β-blockers appeared to be independent protecting factor against higher EATT values. There is an unfavorable positive relationship between ETS exposure estimated using the SHSES scale and EATT in hypertensive patients.

## Introduction

Environmental tobacco smoke exposure (ETS), or passive smoking, continues to be a major health problem worldwide. The global fight against this issue has been going on for years and there is still much to be done. ETS is an inclusive term describing any tobacco smoke exposure outside of active smoking, including second-hand smoke (SHS) and third-hand smoke (THS) [1]. According to the rules set down in the WHO Framework Convention on Tobacco Control, all people should be protected from tobacco smoke. Up to date, 168 countries have signed the Convention and thus agreed on implementing measures to eliminate tobacco smoke exposure in workplaces, public transport and public places. However, despite the fact that recent reports show the increase in implementation of smoke-free laws, only 22% of the world’s population are protected by complete smoking bans in public places [2].

Adverse health effects of tobacco smoke are well-established in medical literature. It has been proven that there is no safe level of exposure [3]. International Agency for Research on Cancer classifies ETS as a “Group 1” human carcinogen [4]. It is harmful for both children and adults. Among children it contributes to ear infections, more frequent and severe asthma attacks, respiratory infections and sudden infant death syndrome (SIDS). Whereas in non-smoking adults it increases the risk for heart disease, lung cancer and stroke [5, 6]. These data stay consistent with statistics, according to a cross-sectional epidemiologic study for 2016, 52.3 individuals who smoked were associated with the death of 1 individual who did not smoke [7]. In 2017, globally, 1.2 million of deaths were attributable to SHS exposure, of which 63,822 occurred among children younger than 10 years old [6].

Many studies indicate that passive smoking is correlated with an increase in blood pressure, especially systolic blood pressure [8–10]. This effect is greatly unfavorable considering the fact that hypertension is a major risk factor for many cardiovascular diseases (CVD), including coronary artery disease and stroke which are leading causes of death worldwide [11, 12]. Moreover, it has been reported that exposure to tobacco smoke not only increases the risk for hypertension but also increases the risk for other cardiovascular diseases in already hypertensive patients [13]. These harmful properties of tobacco smoke are presumably the consequence of endothelial dysfunction. SHS exposure is thought to decrease the endothelium-dependent vasodilatation through inhibition of nitric oxide (NO) synthase and also to cause direct damage to the endothelium [14].

There are many different methods to measure ETS exposure levels; including laboratory measurements and questionnaires [15, 16]. Laboratory methods are divided into two groups: direct and indirect. Direct methods include assessing the amount of biomarker—that is a substance whose level correlates with exposure to a given chemical compound. Nicotine and its metabolite, cotinine, are the most commonly used biomarkers in the assessment of tobacco smoke exposure. Their concentration can be measured in the blood, saliva, urine, milk of nursing mothers or in hair [17, 18]. Indirect methods include measurements of tobacco smoke components in the air, usually in closed spaces such as homes or workplaces. Laboratory methods enable scientists to get precise measurements, however they have many drawbacks. In particular they are expensive and using different analytical methods makes it difficult to compare results between studies. Moreover, numerous variables influence the results, e.g. exposure time or time period between the exposure and testing [16].

ETS exposure level can also be assessed by means of a questionnaire with relevant questions concerning the exposure. Vardavas et al. suggested the use of The Secondhand Smoke Exposure Scale (SHSES) [19]. It is a biomarker validated 11-point scale, which comprises four ranked questions, weighted to each response’s relative contribution to overall nicotine levels among adults. Questions concern the exposure in various places. Although biomarker measurement may be more accurate than a questionnaire, the latter provide affordable and easily accessible ETS assessment.

Epicardial adipose tissue (EAT) is a fat deposit localized between the myocardium and the visceral pericardium. It stays in direct contact with the major coronary arteries and their branches. Previous research has documented that it is a biologically active organ with unique anatomic, biomolecular and genetic properties. EAT is a source of numerous cytokines that act either anti-inflammatory or pro-inflammatory [20, 21]. Imaging methods for EAT include computed tomography (CT), magnetic resonance imaging (MRI), positron emission tomography (PET), echocardiography (ECHO) and nuclear medicine techniques [20]. Nowadays, many research indicate that EAT measurements can serve as a modifiable risk factor and additional tool for cardiovascular risk stratification [21, 22]. It has also been reported that EAT accumulation is larger among hypertensive than normotensive patients. Moreover, some scientists indicate that EAT measurements could be useful for identification of hypertensive patients and prediction of hypertension severity [22].

Due to the fact that both exposure to tobacco smoke and the activity of epicardial adipose tissue seem to impact coronary arteries we have decided to assess the relationship between them.

The aim of the study was to assess the relationship between environmental tobacco smoke exposure (ETS) estimated using the SHSES scale and epicardial adipose tissue thickness (EATT) in hypertensive patients.

## Materials and Methods

A total of 96 hypertensive patients were recruited for this study. The group consisted of 48 females and 48 males with the mean age of 69.32 ± 9.54 years (age range: 38–87 years). The inclusion criteria were as follows: age ≥ 18, hypertension diagnosed and pharmacologically treated for at least 5 years, indication to coronary computed tomography angiography and no history of smoking cigarettes. Patients with secondary hypertension, previously diagnosed ischemic heart disease, previous stroke, type 2 diabetes, hyperthyroidism or hypothyroidism, chronic kidney disease and patients with insufficient quality of the coronary computed tomography angiography were excluded from the study. The clinical characteristics of the studied group of patients are presented in Table 1.

**Table 1:** Clinical characteristics of the studied group

Number^a^	100.0
Age (years)^b^	69.32 ± 9.54
Gender	
Men^a^	50.0
Women^a^	50.0
Height (cm)^b^	167.73 ± 8.16
Body mass (kg)^b^	73.11 ± 11.96
BMI (kg/m^2^)^b^	26.91 ± 3.35
Overweight^a^	48.9
Obesity^a^	17.7
Essential hypertension^a^	100.0
Systolic blood pressure (mmHg)^b^	139.17 ± 15.33
Diastolic blood pressure (mmHg)^b^	87.52 ± 10.04
Grades of hypertension	
Mild^a^	51.0
Moderate^a^	46.9
Severe^a^	2.1
Hypotensive drugs	
ACE inhibitors^a^	52.1
β-blockers^a^	44.8
Diuretics^a^	27.1
Calcium channel blockers^a^	31.2
Angiotensin receptor blockers^a^	17.9
Other hypotensive drugs^a^	1.0
Lipid profile	
Total cholesterol (mg/dl)^b^	222.61 ± 50.43
LDL cholesterol (mg/dl)^b^	114.38 ± 27.15
HDL cholesterol (mg/dl)^b^	56.10 ± 17.35
Triglycerides (mg/dl)^b^	179.30 ± 148.31
Fasting glucose (mg/dl)^b^	107.85 ± 36.84

*ACE* angiotensin-converting enzyme, *BMI* body mass index, *HDL* high density lipoprotein, *LDL* low density lipoprotein

^a^Percentages

^b^Arithmetic mean ± standard deviation

The methodology included medical history questionnaire, basic anthropometric measurements, blood pressure measurements, determination of total cholesterol, LDL cholesterol, HDL cholesterol, triglycerides and fasting glucose in blood, questionnaire assessment of tobacco smoke exposure and coronary computed tomography angiography (CCTA).

Blood pressure values were measured by the Korotkov method. Mild hypertension was diagnosed in patients with systolic blood pressure (SBP) between 140 and 159 mmHg and/or diastolic blood pressure (DBP) between 90 and 99 mmHg. Moderate hypertension was diagnosed in patients with SBP between 160 and 179 mmHg and/or DBP between 100 and 109 mmHg. Severe hypertension was diagnosed in patients with SBP ≥ 180 mmHg and/or DBP ≥ 110 mmHg. Blood cholesterols, triglycerides and glucose concentration were determined by standard methods according to the instructions of the manufacturer of the used reagent kits.

Environmental tobacco smoke exposure was assessed with SHSES according to Vardavas et al. [19]. SHSES sheet contains four questions, each relating to different site of exposure. Namely, exposure at home per day, exposure in a car per day, exposure in public places the past week and exposure at work the past week. Every answer is assigned to a certain amount of points. It enables a quantitative assessment of ETS exposure. The maximum score of the SHSES is 11 points and the minimum score is 0. SHSES and its interpretation is presented in Table 2.

**Table 2:** The Secondhand Smoke Exposure Scale (SHSES) and its interpretation [19]

	Score
Exposure at home per day	
> 20 cigarettes per day	5
10–20 cigarettes per day	4
6–9 cigarettes per day	3
1–5 cigarettes per day	2
None	0
Exposure in a the car per day	
30 min or more	3
Less than 30 min	2
Never	0
Exposure in public places the past week	
Once or more	2
Never	0
Exposure at work the past week	
Once or more	1
Never	0
Total SHSES score	0–11
No ETS exposure	0
Low ETS exposure	1–3
Medium ETS exposure	4–7
High ETS exposure	8–11

*ETS* environmental tobacco smoke

**Fig. 1:**
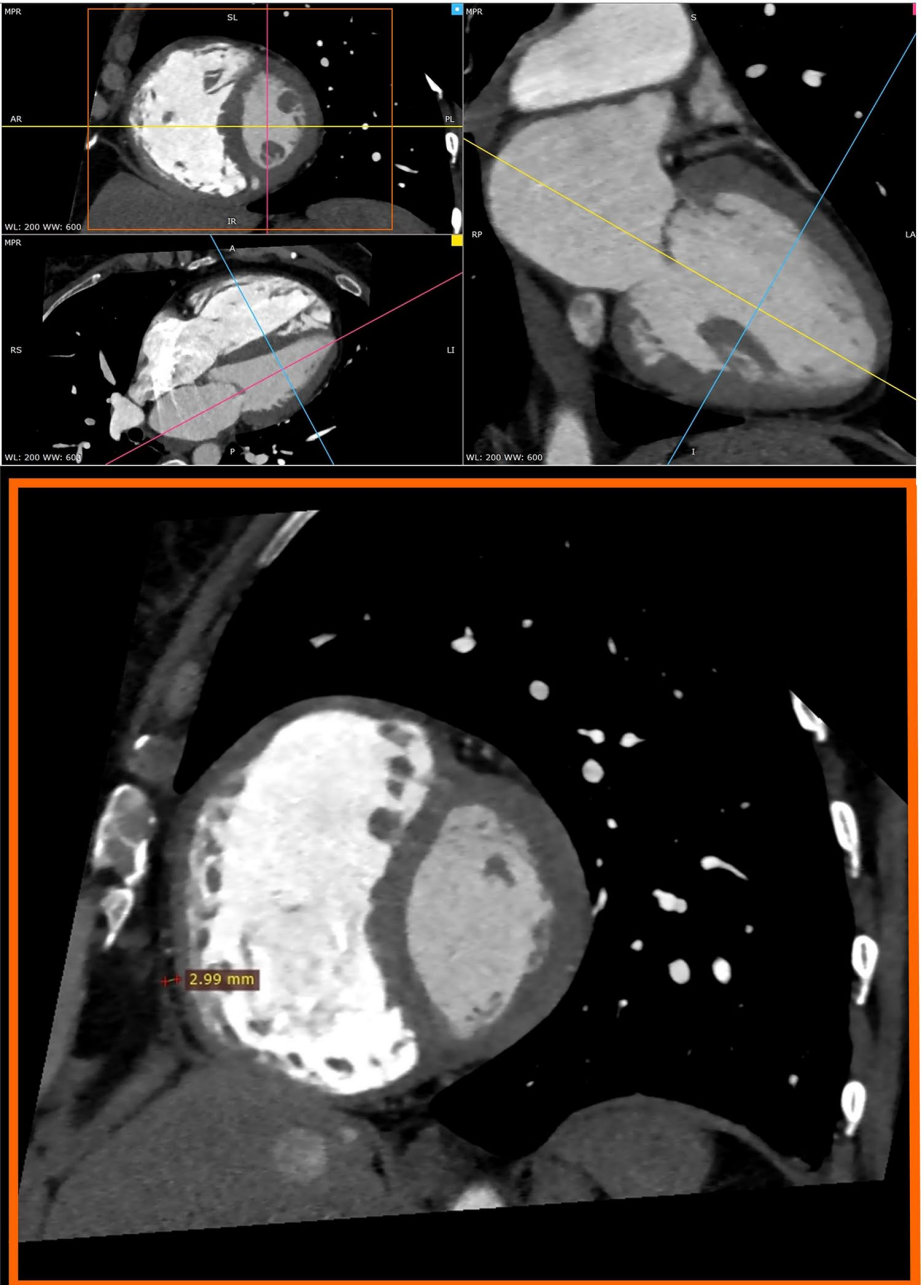
Assessment of the epicardial adipose tissue thickness in computed tomography angiography images

The coronary computed tomography angiography was performed using the standard protocol and dual source 128-slice CT scanner SOMATOM Definition Dual-Source CT (Siemens Healthcare, Germany). The test protocol included the following stages: topogram, phase without applying intravenous contrast agent intended to estimate the coronary artery calcium score (CACS), bolus tracking, administering nitroglycerin, phase with intravenous contrast agent intended for proper assessment of the heart and coronary arteries. Iodine, non-ionic contrast agent—Iomeprol (Iomeron 400, Bracco UK Ltd, Great Britain), was used, administered intravenously using automatic syringe through the veins of the cubital fossa. Assessment of the EATT was made using an application for post-processing of computed tomography images syngo.CT Cardiac Function (Siemens Healthcare, Germany). EATT was measured on the basis of multiplane reconstruction (MPR) along the short axis of the left ventricle, at the level of mid-ventricular slices. Maximal thickness was measured from the myocardial wall to the pericardium, over the right ventricular free wall. The final EATT values were deemed the average from independent assessments made by two radiologists with several years’ experience in the assessment of computed tomography examinations. An example of EATT measurement is shown in Fig. 1.

The results obtained in the SHSES questionnaire were used to divide patients into subgroups differing in ETS exposure. In the basic comparison, the subgroups were as follows: subgroup A—no ETS exposure (SHSES = 0 points, *n* = 48), subgroup B—low ETS exposure (SHSES = 1–3 points, *n* = 11), subgroup C—medium ETS exposure (SHSES = 4–7 points, *n* = 20) and subgroup D—high ETS exposure (SHSES = 8–11 points, *n* = 17). In addition, subgroups based on the answers to each question of the SHSES questionnaire were distinguished.

Ethical approval from the Local Ethics Committee was obtained. All the study participants provided written informed consent.

Statistical analyses were performed using a statistical package “Dell Statistica 13” (Dell Inc., USA). Quantitative variables were presented as arithmetic means ± standard deviations. The distribution of variables was tested using Lilliefors test and Shapiro–Wilk test. In the case of independent quantitative variables with a normal distribution, the *t* test or the ANOVA variance analysis were used. In the case of independent quantitative variables with a distribution other than normal, the Mann–Whitney *U* test or the ANOVA Kruskal–Wallis test was used. Results for qualitative variables were expressed as percentage values. For independent qualitative variables, the Chi-square test was used. To determine the relationships between the studied variables, correlation and regression analyses were carried out. Results at the level of *p* < 0.05 were considered to be statistically significant.

## Results

Within the study group the mean SHSES score was 3.05 ± 3.74. According to the SHSES score 50.0% subjects were not exposed to ETS, and another 50.0% were exposed to ETS. In whole study group 11.5% had low ETS exposure level, 20.8% medium ETS exposure level and 17.7% high ETS exposure level. 14.6% of patients were exposed to ETS from ≥ 10 cigarettes smoked by other household members per day. Of all subjects, 35.4% were exposed to ETS at home daily, 34.4%—in the car daily. 40.6% were exposed to ETS in public places once or more the past week, and 27.1% at work once or more the past week.

The mean EATT in whole study group was 5.75 ± 1.85 mm. EATT was statistically significantly higher in subgroup D (high ETS exposure) than in subgroups A and B (no ETS exposure and low ETS exposure) (A: 5.28 ± 1.64 mm, B: 5.04 ± 2.64 mm, D: 7.04 ± 2.64 mm, *p*_A–D_ and *p*_B–D_ < 0.05). The average EATT value for subgroup not exposed to ETS was statistically significantly higher than for subgroup exposed to ETS (5.28 ± 1.64 mm vs. 6.22 ± 1.94 mm, *p* < 0.05).

The analysis of the relationship between EATT and the exposure in different sites was performed for each of the four locations listed in the SHSES scale. In subgroup exposed to ETS at home daily, EATT measurement was statistically significantly higher when compared with subjects not exposed to ETS at home (6.67 ± 1.66 mm vs. 5.24 ± 1.76 mm, *p* < 0.05). In subgroup exposed to ETS in the car daily, EATT was also statistically significantly higher than in patients who were not exposed (6.78 ± 1.46 mm vs. 5.21 ± 1.81 mm, *p* < 0.05). Moreover, in subgroup exposed to ETS in public places the past week, EATT was statistically significantly higher than in subjects who were not exposed (6.56 ± 1.60 mm vs. 5.19 ± 1.81 mm, *p* < 0.05). There was no statistically significant difference in EATT between subgroup exposed to ETS at work the past week and subgroup not exposed to ETS at work the past week (6.28 ± 2.38 mm vs. 5.55 ± 1.58 mm, *p* > 0.05). EATT in subgroups divided based on ETS exposure is presented in Table 3.

**Table 3:** EATT in subgroups divided based on ETS exposure

Differentiation criterion	Subgroup	EATT (mm)^a^	*p* value
SHSES score	A. No ETS exposure (*n* = 48)	5.28 ± 1.64	A. vs. D.: < 0.001 B. vs. D.: < 0.01
	B. Low ETS exposure (*n* = 11)	5.04 ± 2.64	
	C. Medium ETS exposure (*n* = 20)	6.17 ± 1.18	
	D. High ETS exposure (*n* = 17)	7.04 ± 2.64	
SHSES score	E. No ETS exposure (*n* = 48)	5.28 ± 1.64	E. vs. F.: < 0.05
	F. ETS exposure (*n* = 48)	6.22 ± 1.94	
Exposure at home per day	G. > 20 cigarettes per day (*n* = 3)	9.64 ± 0.69	G. vs. H, I, J.: < 0.01 G. vs. K.: < 0.001 H, I vs. K.: < 0.05
	H. 10–20 cigarettes per day (*n* = 11)	6.62 ± 9.64	
	I. 6–9 cigarettes per day (*n* = 16)	6.27 ± 1.24	
	J. 1–5 cigarettes per day (*n* = 4)	6.20 ± 2.00	
	K. None (*n* = 62)	5.24 ± 1.76	
Exposure at home per day	L. Yes (*n* = 34)	6.67 ± 1.66	L. vs. M.: < 0.001
	M. No (*n* = 62)	5.24 ± 1.76	
Exposure in the car per day	N. 30 min or more (*n* = 8)	7.87 ± 1.97	N. vs. O.: < 0.05 N. vs. P.: < 0.001 O. vs. P.: < 0.01
	O. Less than 30 min (*n* = 26)	6.43 ± 1.10	
	P. Never (*n* = 63)	5.21 ± 1.81	
Exposure in the car per day	Q. Yes (*n* = 33)	6.78 ± 1.46	Q. vs. R.: < 0.001
	R. No (*n* = 63)	5.21 ± 1.81	
Exposure in public places the past week	S. Once or more/yes (*n* = 39)	6.56 ± 1.60	S. vs. T.: < 0.001
	T. Never/no (*n* = 57)	5.19 ± 1.81	
Exposure at work the past week	U. Once or more/yes (*n* = 26)	6.28 ± 2.38	U. vs. W.: ns
	W. Never/no (*n* = 70)	5.55 ± 1.58	

*EATT* epicardial adipose tissue thickness, *ETS* environmental tobacco smoke, *SHSES* Secondhand Smoke Exposure Scale, *ns* non-statistically significant

^a^Arithmetic mean ± standard deviation

**Fig. 2:**
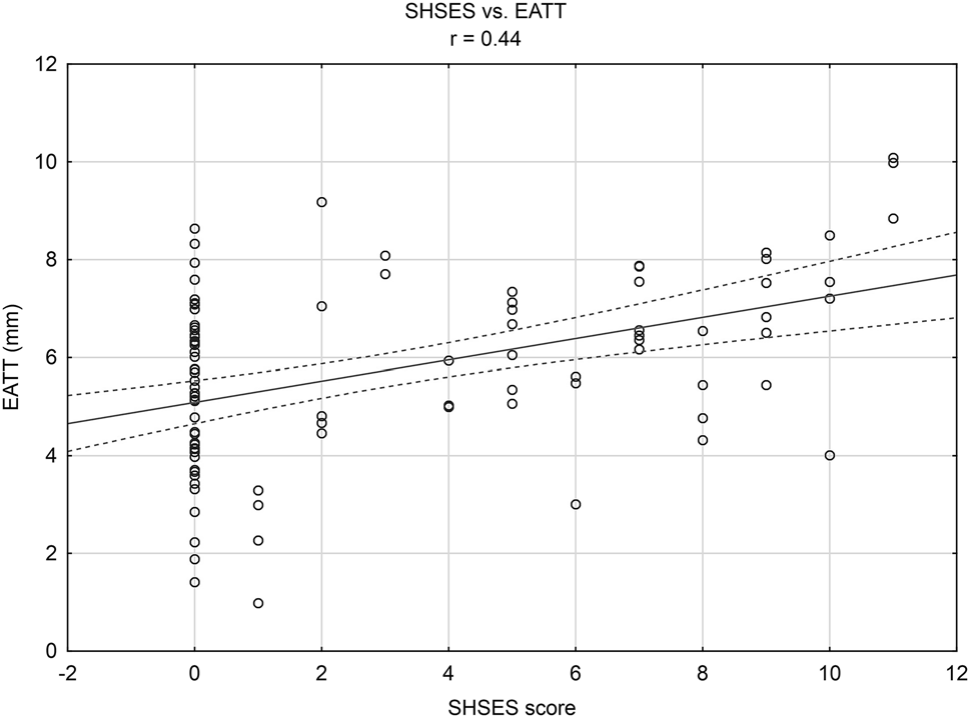
Correlation between the exposure to environmental tobacco smoke expressed by the number of points on the SHSES scale and epicardial adipose tissue thickness

There was a positive statistically significant correlation between the exposure to ETS expressed by the number of points on the SHSES scale and EATT (*r* = 0.44, *p* < 0.05), Fig. 2. A positive statistically significant correlation between the exposure to ETS expressed by the number of points on the SHSES scale and EATT was demonstrated both in the subgroup of patients with mild hypertension (*r* = 0.40, *p* < 0.05) and in the subgroup of patients with moderate hypertension (*r* = 0.46, *p* < 0.05). Due to the size of the subgroup (*n* = 2), the above correlation in patients with severe hypertension could not be tested. In addition, positive linear correlation have been demonstrated between: body mass index (BMI) and EATT (*r* = 0.35, *p* < 0.05), systolic blood pressure and EATT (*r* = 0.36, *p* < 0.05), as well as diastolic blood pressure and EATT (*r* = 0.32, *p* < 0.05).

The multivariable stepwise backward regression analysis was used to define the relationships between basic body parameters (age, gender, BMI), characteristics of hypertension (systolic and diastolic blood pressure, grades of arterial hypertension, hypotensive treatment), basic biochemical parameters (lipid profile parameters and fasting glucose), exposure to ETS and EATT. Regression analysis yielded that higher SHSES score, higher BMI, higher systolic blood pressure and higher diastolic blood pressure are independent risk factors for higher EATT values. On the contrary, the use of ACE inhibitors and β-blockers appeared to be an independent factor protecting against higher EATT values. The results of estimation for significant model obtained through regression analysis are presented in Table 4.

**Table 4:** Results of estimation for the final model obtained in multivariable stepwise backward regression analysis

	Model for: EATT (mm)			
	Regression coefficient	SEM of regression coefficient	*p*	
BMI (kg/m^2^)^a^	0.062	0.027	< 0.05	*p* < 0.001 *R*^2^ = 0.774 SEM of the model: ± 1.013
Systolic blood pressure (mmHg)^a^	0.021	0.009	< 0.05	
Diastolic blood pressure (mmHg)^a^	0.063	0.014	< 0.001	
ACE inhibitors^b^	− 1.153	0.230	< 0.001	
β-blockers^b^	− 1.100	0.229	< 0.001	
SHSES score^a^	0.107	0.029	< 0.01	

*ACE* angiotensin-converting enzyme, *BMI* body mass index, *EATT* epicardial adipose tissue thickness, *SHSES* Secondhand Smoke Exposure Scale

^a^Quantitative variables

^b^Dichotomic variables, where 1: yes, 0: no

## Discussion

Our study showed that in patients with essential hypertension there is an unfavorable positive relationship between the exposure to ETS expressed by the number of points on the SHSES scale and EATT. This provides a good starting point for discussion and further research on the pathomechanism for this relationship.

Epicardial adipose tissue has received much attention in the past decade [20, 21]. Many researchers strive to understand its unique function and establish its influence on cardiovascular diseases (CVDs). EAT has no fascia layer separating it from the underlying myocardium which indicates that these two structures share the same microcirculation and thus are closely related. Up to date, it is known that EAT acts comprehensively and has mechanical, metabolic, thermogenic, and endocrine/paracrine functions [23, 24]. Under normal physiological conditions, EAT serves as a donor of free fatty acids (FFA), which are an immediate ATP source for the myocardium, and produces anti-inflammatory compounds, such as adiponectin and adrenomedullin. However, under pathological conditions, EAT releases inflammatory adipokines, such as resistin and leptin, and has an adverse lipotoxic effect which contributes to increased risk for CVDs [23].

Numerous studies have reported the relationship between EAT and the cardiovascular health. Yang Lu et al. has recently reported that both large EAT volume and high EAT density were associated with cardiac structure and function in patients with no coronary artery disease. In the study, cardiac geometry was distorted in people with larger EAT deposits, inter alia, by increased interventricular septal thickness and left ventricular mass [25]. These results stay consistent with the articles previously published on the subject [26, 27]. Similarly, studies on patients with coronary artery disease yield a correlation between EAT thickness and the severity of coronary artery disease (CAD). Mazurek et al. compared the proinflammatory activity in epicardial adipose tissue with subcutaneous adipose tissue in patients with CAD [28]. Interestingly, the study demonstrated increased inflammatory responses in EAT in patients with significant CAD. These patients had significantly higher levels of proinflammatory cytokines such as IL-1β, IL-6, MCP-1, and TNF-α in EAT deposits. Moreover, these changes were noted irrespective of clinical variables (diabetes, body mass index, and chronic use of statins or ACE inhibitors/angiotensin II receptor blockers) or plasma concentrations of circulating biomarkers. Recent studies seem to confirm these findings and also indicate that EAT is linked to early coronary plaque formation and suggest that routine assessment of EAT could be implemented for a better prediction and stratification of CAD [29–31].

To our knowledge there are no studies assessing the impact of passive smoking assessed by the SHSES on EATT. However, similar relationship has been found in patients actively smoking cigarettes. Monti et al. suggested that, in subjects with metabolic syndrome (MeS), cigarette smoking is an independent predictor of increased epicardial fat volume and of more advanced changes in coronary vessels expressed as higher calcium score (CS) [32]. This view is supported by molecular studies of cytokines produced by EAT. Mach et al. conducted a cross-sectional study to assess tissue concentrations of tumor necrosis factor-ɑ (TNF-α), interleukin-6 (IL-6), adipocyte fatty acid-binding protein, leptin, and adiponectin in adipose tissue, namely in EAT and subcutaneous deposits (SAT), in smokers, non-smokers and former smokers. The results showed that smoking was independently associated with higher TNF-α and IL-6 concentrations in both EAT and SAT. In addition, former smokers had their cytokines levels lower than smokers which highlights the importance of smoking cessation on decreasing inflammation and thus improving cardiac health [33]. It can be concluded that both active smoking and passive smoking cause unfavorable changes in epicardial adipose tissue. Moreover, according to our results, the risk seems to increase with the level of exposure.

Our study showed that higher systolic and diastolic blood pressure are independent risk factors for higher EATT values. In addition, we also found that the use of ACE inhibitors and β-blockers contributes to lower EATT values. These results are in line with previous studies. In a study by Özdil et al. EAT thickness measured by echocardiography was an independent predictor of new diagnosis of hypertension [34]. Chang et al. performed a study on 214 non-obese patients hospitalized with acute or chronic non-cardiogenic illness and found that EAT thickness was independently correlated with hypertension, age, and BMI [35]. Moreover, in another study on 80 patients who were diagnosed with hypertension EAT thickness showed a significant correlation with 24-h SBP (*r* = 0.434, *p* < 0.001) [36]. Nevertheless, the exact mechanism of this correlation requires additional research and the issue whether the EAT is an independent risk factor for hypertension or just coexists as a result of mutual risk factors is yet to be determined.

We are aware that our research has some limitations. In terms of material, the main limitation of the study is the relatively small number of patients included in the study. Another limitation is the lack of information about menopause women included in the study. In terms of methodology, first of all, to assess the environmental smoke exposure we have used a SHSE scale which is based on respondents' answers. It always carries the risk that participants would withhold some information and under- or overestimate the result. However, we believe that complete information about the importance of the study and providing the right environment made the answers reliable. In our opinion the advantage of the scale is that it is easy to use and constitutes a biomarker-validated approach. According to Vardavas et al. the correlation between the SHSES and the participants biomarker validated exposure (hair nicotine), indicated a Pearson’s correlation of 0.4939, with a *p*-value < 0.0001 [19]. Second of all, we have decided to measure EAT thickness by means of multislice computed tomography. Even though research indicates that it is more accurate than echocardiography; it is the cardiac magnetic resonance that is considered a gold standard [37]. Nevertheless, according to the literature on EAT measurements, coronary computed tomography angiography has a reasonable degree of accuracy when compared to cardiac magnetic resonance and is undoubtedly more accessible [38]. It should also be considered that there are some new parameters that are used to assess the EAT deposits, such as EAT volume and density [39–41]. Further work needs to be done to establish whether they are correlated with ETS exposure.

Taken together, this study suggests that in patients with essential hypertension, there is a positive relationship between environmental tobacco smoke exposure estimated using the SHSES scale and EATT. In addition, we also found that higher BMI and higher blood pressure values are associated with higher EATT, and the use of ACE inhibitors and β-blockers contributes to lower EATT. These findings provide additional information on the effects of tobacco smoke exposure on cardiovascular health. Future research should further develop and confirm these initial findings.
